# Effect of the Natural Product Triptolide on Pancreatic Cancer: A Systematic Review of Preclinical Studies

**DOI:** 10.3389/fphar.2017.00490

**Published:** 2017-08-25

**Authors:** Chi Zhang, Xiao-Juan He, Li Li, Cheng Lu, Ai-Ping Lu

**Affiliations:** ^1^Institute of Basic Research in Clinical Medicine, China Academy of Chinese Medical Sciences Nanxiaojie, Beijing, China; ^2^School of Chinese Medicine, Hong Kong Baptist University Kowloon Tong, Hong Kong

**Keywords:** triptolide, pancreatic cancer, biochemical mechanisms, systematic review

## Abstract

**Objective:** Triptolide (TL), a natural product isolated from Tripterygium wilfordii Hook F (TwHF), shows potent anticancer effects *in vitro* and *in vivo*. We aimed to summary the biochemical mechanisms through which TL has been shown to induce apoptosis, autophagy and inhibit angiogenesis in pancreatic cancer (PC).

**Methods:** We undertook a systematic review of preclinical evidence. We searched electronic databases. The potential mechanisms and the underlying signaling pathways have been accumulated as well.

**Results:** We screened 116 abstracts for eligibility and included 17 preclinical studies in the analysis. Details of the animals and cell lines were provided in 16 studies (94.1%). Six studies (75.0%) randomly assigned animals to treatment or control groups and only 1 study (12.5%) reported on blinding. The majority of the TL's research field has focused on its pro-apoptotic effects through downregulation of inhibitory pathways and upregulation of apoptotic pathways. The studies have shown that TL is effective both *in vitro* and *in vivo* against PC cells.

**Conclusion:** This study provides a systematic summary of TL's anti-pancreatic cancer profile and the underlying mechanisms of with special emphasis on the apoptosis, autophagy, angiogenesis and related molecular pathways. Improved study quality in terms of treatment allocation methods reporting, randomization and blinding will accelerate needed progress toward trials.

## Introduction

Pancreatic cancer (PC) is a highly lethal malignancy with few effective treatments (Manuel, 2010). Only 10–20% of patients receive a diagnosis at a stage that is amenable to surgical resection and possible cure (Michalski et al., 2007). Gemcitabine, which works as a nucleoside analog for deoxycytidine triphosphate, is used as a first line treatment for PC (Di et al., 2010). However, like any chemotherapy, chemoresistance is the major impediment for treating PC (Rivera et al., 2009). Undoubtedly, novel treatment strategies are clearly needed. Many potential treatments are being tested in the preclinical stage. One in particular, a natural product called triptolide (TL), has shown promise as an anticancer drug for many different types of cancer, including PC (Wang et al., 2016). TL is an extract from the plant Tripterygium wilfordii Hook F (TwHF), also known as the thunder god vine or lei gong teng in Chinese (Kupchan et al., 1972). TwHF grows widely in the mountainous regions of southeast and southern China and has been used as a novel immunosuppressive and anti-inflammatory agent, particularly for treating rheumatoid arthritis (RA) (Lv et al., 2015). The biological activities of TwHF have been broadly investigated, as evidenced by the more than 54,152 and 1,116 publications (related to this herb) that are available in the China National Knowledge Infrastructure (CNKI) Databases and PubMed, respectively.

**Figure 1:**
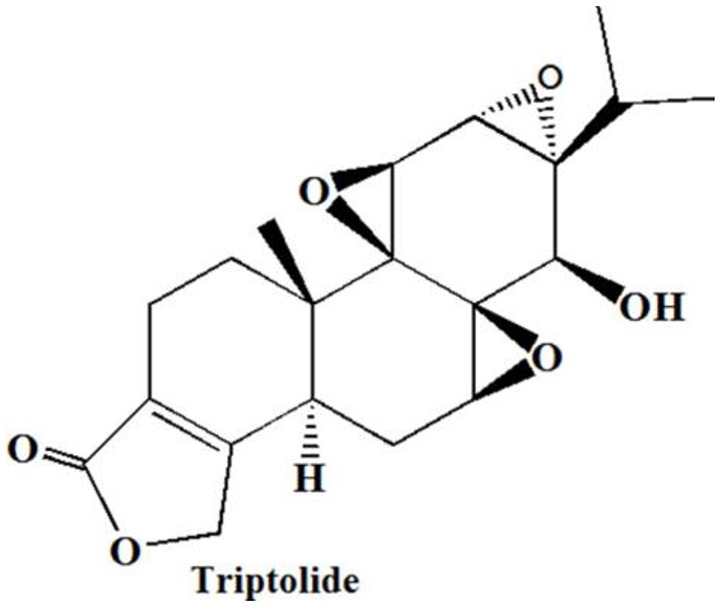
Chemical structures of TL (Wang et al., 2016).

TL is a diterpenoid triepoxide that was isolated from the roots of TwHF in 1972 (Figure 1; Kupchan et al., 1972; Wang et al., 2016). Although TL has not undergone extensive clinical testing, it shows broad spectrum anticancer effects, including proliferation inhibition, apoptosis induction and metastasis inhibition. TL plays its anticancer roles through inhibited and activated biochemical mechanisms (Manzo et al., 2012). There is a need to briefly summarize these mechanisms, particularly to focus on the pathways.

This systematic review aims to generate preclinical data on the effect of TL on PC, summarize the biochemical mechanisms through which TL has been shown to induce apoptosis and autophagy and inhibit angiogenesis in PC, evaluate the quality of prior studies (in term of treatment allocation methods, reporting, randomization and blinding), and investigate how recent advancements in TL could bring it to the forefront of chemotherapy for PC.

## Methods

### Information sources and search strategy

The systematic review was in line with recommendations from the Preferred Reporting Items for Systematic Reviews and Meta-Analyses (PRISMA) statement. Pubmed, Embase, China National Knowledge Infrastructure (CNKI), Wanfang, and Sionmed were searched using the search terms “Triptolid,” “Minnelide (a water-soluble prodrug of triptolide),” “pancreatic cancer.” The search covered the period from their inception (Pubmed 1966, Embase 1974, CNKI 1979, Wanfang 1960, and Sionmed 1978) to 01 March 2017. No language restriction was applied. All relevant references were checked for additional and unpublished citations.

### Eligibility criteria, study selection, and data collection

Preclinical studies that have investigated the effect of TL on PC fulfilled the inclusion criteria. Only full publications were considered. All studies were assessed by two investigators (CZ and XJH). When a study was reported in several publications, we analyzed only the most complete set of data to avoid double counting cases. Two authors independently reviewed the articles obtained from the search. To ascertain the extent to which TL can impart its anticancer effects upon PC cells, we investigated which pathways and mechanisms are affected by it. A comparison was performed, and discrepancies were resolved through dialog.

## Results

### Study characteristics

After screening 116 citations, we reviewed 66 potentially relevant full-text articles and identified 17 primary publications. Of these, there were 16 English language papers and 1 was published in Chinese. Eight studies (47.1%) with had both *in vitro* and *in vivo* assessments (Table 1) provided explicit information in the methods section concerning the number of animals allocated to each treatment group, and they accounted for all animals in their results. Six animal studies (75.0%) randomly assigned animals to treatment groups or control groups, and one study (12.5%) reported that it used a blinded pathologist (Phillips et al., 2007). Details of the animals and cell lines (i.e., the source) were provided in 16 studies. One study that used an animal model (athymic mice) was extremely detailed (Wang et al., 2012). None of the studies specified or provided a power calculation or sample size determination. The results listed the main characteristics of the relevant pathways and included references.

**Table 1:** Summary of 17 preclinical studies identified.

**References**	***In vitro*/*in vivo***	**Cell culture/Disease model**	**Endpoint studied**	**Animal allocation and Flow described**	**Randomized**	**Blinded**	**Main findings**
Banerjee et al., 2013	Both	MIA-PaCa2 was obtained from the ATCC. Female nude mice (4–6 weeks old) were used (Charles River Laboratories).	To study the effect of OGT inhibition on PC cells.	Yes	Yes	Unclear	The authors observed that down-regulation of Sp1 by mithramycin, siRNA, TL resulted in decreased NF–κB activity in both cell lines and animal models.
Chen et al., 2013	Both	MIA PaCa-2 cells derived from a primary pancreatic tumor were obtained from Ascites-derived AsPC-1. Three de-identified human tumors were implanted subcutaneously into SCID animals (Jackson Laboratory).	Mcl-1 and miR-204 expression in PC cells *in vitro* and *in vivo*.	Yes	Unclear	Unclear	TL mediated miR-204 increase causes PC cell death via loss of Mcl-1.
Wang, 2012	*In vitro*	ASPC-1 was purchased from the ATCC.	To detect the expression changes of PARP and caspase-3.	NA	NA	NA	The proliferation of AsPC-1 cells was inhibited by TL.
Borja-Cacho et al., 2010	*In vitro*	MIA-PaCa2 and PANC-1 cells were obtained from the ATCC.	To explore cell viability, apoptosis, caspase-3 and caspase-9 activities, and poly(ADP)-ribose polymerase cleavage.	NA	NA	NA	TL and TNF-related apoptosis-inducing ligand decreased the cell viability in all the cell lines and increased apoptotic cell death as a result of caspase-3 and caspase-9 activation.
Ding et al., 2015	Both	The PANC1, ASPC1 and SW1990 were purchased from the ATCC (Rockville, MD, USA). Athymic nude mice (5-weekoldfemales) were purchased from Shanghai Laboratory Animal Center of Chinese Academy of Science (Shanghai, China).	To measure expression of HIF-1α and VEGF.	Yes	Unclear	Unclear	The expression of HIF-1α was shown to be reduced in pancreatic cell lines following treatment with TL.
Dudeja et al., 2009	*In vitro*	Panc-1 and MiaPaCa-2 were a kind gift from Dr Edward E. Whang (Brigham and Women's Hospital, Harvard Medical School, Boston, MA).	To measure intracellular Ca2, cytosolic cathepsin B activity, caspase-3 activity, cell viability, and lysosome integrity.	NA	NA	NA	HSP70 expression was down-regulated in cultured PC cells by exposure to TL.
Liu and Cui, 2013	Both	PANC-1, CFPAC-1 and normal pancreatic cell line HPC-Y5, were obtained from the ATCC (Manassas, VA, USA). Five-week-old male BALB/c athymic nude mice were obtained from Shanghai Laboratory Animal Center of Chinese Academy of Sciences.	*In vitro*, cell viability and apoptosis were analyzed. *In vivo*, inhibitory effects of TL were assessed.	Yes	Yes	Unclear	The combination Of tl and artesunate can exert synergistic anti-tumor effects in PC cells.
Ma et al., 2013	Both	PANC-1 was obtained from the ATCC (Manassas, VA, USA) Four- to 6-week-old BALB/c nude mice were obtained from Shanghai SLAC Laboratory Animal Co., Ltd. (Shanghai, China).	To measure cell viability and apoptosis, and to examine the expression of COX-2 and VEGF.	Yes	Yes	Unclear	TL induces apoptosis and inhibits proliferation of PANC-1 PC cells by downregulating COX-2 and VEGF.
Mackenzie et al., 2013	Both	MIA PaCa-2, Capan-1, and HEK-293 lines were obtained from the ATCC. Female SCID mice were used (The Jackson Laboratory, Bar Harbor, ME).	To measure miR-142-3p and HSPA1B (HSP70) levels.	Yes	Yes	Unclear	TL downregulated HSP70, and induced the expression of miR-142-3p.
Mujumdar et al., 2010	*In vitro*	MIA-PaCa2 and PANC-1 cells were obtained from the ATCC.	To measure viability of PC cells, and to examine Caspase-3, Atg5, and Beclin1 levels.	NA	NA	NA	TL kills PC cells by 2 different pathways. It induces caspase-dependent apoptotic death in Mia- PaCa-2, Capan-1, and BxPC-3, and induces caspase independent autophagic death in S2-013, S2-VP10, and Hs766T.
Mujumdar et al., 2014	*In vitro*	MIA PaCa-2 was obtained from the ATCC.	To investigate the GRP78 expression in PC cells.	NA	NA	NA	TL kills PC cell lines by inducing the UPR, resulting in ER stress by inhibiting expression of the survival protein GRP78.
Phillips et al., 2007	Both	MIA-PaCa2 and PANC-1 cells were obtained from the ATCC. Female nude mice (4–6 weeks old) were used (Charles River Laboratories).	To examine the effects of TL on (a) PC cells by assessing viability and apoptosis, (b) PC growth and local invasion *in vivo*, and (c) HSP70 levels in PC cells.	Yes	Yes	Yes	TL causes PC cell death by the induction of apoptosis, and its mechanism of action is mediated via the inhibition of HSP70.
Qiao et al., 2016	*In vitro*	BxPC.-3 and PANC.-1 cell lines were obtained from the Shanghai Institute of Biochemistry and Cell Biology, Chinese Academy of Sciences. (Shanghai, China).	To investigate the CHK1 expression in PC cells.	NA	NA	NA	TL synergistically increased gemcitabine–induced cell growth inhibition and apoptosis, in addition to the cooperative regulation of B–cell lymphoma2 family proteins and loss of mitochondrial membrane potential.
Wang et al., 2006	*In vitro*	HeLa (CCL-2), PANC-1 (CRL-1469), and RAW264.7 (TIB-71) cells were purchased from ATCC (Manassas, VA, USA).	To examine the effect of TL on the growth of pancreatic carcinoma PANC-1 and cervical adenocarcinoma HeLa cells.	NA	NA	NA	The authors found that TL potently induces apoptosis through activating the caspase cascade associated with Bid cleavage.
Wang et al., 2012	Both	AsPC-1, MiaPaCa-2, BxPC-3, PANC-1 and SW480 were obtained from ATCC (Manassas, VA, USA). Athymic mice without detailed descriptions.	To examine the effect of TPL on cell growth inhibition and apoptosis of PC cells *in vitro* and *in vivo*, and further explore the regulatory role of DcR3 in TPL-induced apoptosis pathway.	Yes	Yes	Unclear	TPL inhibited the proliferation and induced the apoptosis of PC cells, also inhibited DcR3 expression.
Zhou et al., 2007	*In vitro*	ASPC-1, PANC-1 and SW1990 were purchased from the ATCC (Rockville, MD).	To investigate the effects of TL on proliferation and apoptosis of PC cells.	NA	NA	NA	TL significantly down-regulated 5-lipoxygenase (5-LOX) expression, as well as downstream leukotriene B4 (LTB4) production, in PC cell lines.
Zhou et al., 2008	*In vitro*	SW1990 was obtained from Professor Wang Xing Peng (Shanghai, China).	To investigate the inhibitory effects of TL on apoptosis and angiogenesis of PC.	NA	NA	NA	TL induced the apoptosis in human PC cells by up-regulating the expression of apoptosis associated caspase-3 and bax gene.

*ATCC, American Type Culture Collection; COX-2, cyclooxygenase-2; NA, not applicable; PC, pancreatic cancer; SCID, severe combined immunodeficient; TL, TL; VEGF, vascular endothelial growth factor*.

#### Apoptosis pathways

The proapoptotic effects of TL start at the level of competitive decoy receptor 3 (DcR3) binding. DcR3 is downregulated by TL in PC cells (Wang et al., 2012). As a result, more Fas ligand (FasL) will be available for binding to Fas receptor (FasR), increasing the strength of pro-apoptotic signaling. Furthermore, TL can upregulate FADD and FasL, thereby producing a powerful apoptotic signal (Wang et al., 2012).

Differing from the straightforward manner in which TL affects the Fas-mediated pathway, the intrinsic apoptosis pathway is influenced by numerous up- and downregulations. For example, other pro- and anti-apoptotic proteins, the most prominent of which is the Bcl-2 family, modulate the sensitivity of cancer cells to apoptosis (Dudeja et al., 2009). Bid, one of the Bcl-2 proteins, is activated by the actions of TL on the Fas-mediated pathway (Wang et al., 2006). However, the mechanism of Bid cleavage is unclear (Wang et al., 2006).

Furthermore, the expression of BAX is upregulated in PC cells through TL treatment (Zhou et al., 2008) and an increased level of cytochrome c in the cytosol (Wang et al., 2012). These two findings suggest that the activation of tBid through TL may indicate that TL plays a role in cytochrome c release through the mitochondrial apoptosis-induced channel. TL's activation of tBid is not the only mechanism through which it alters the permeability of the mitochondrial membrane to promote apoptosis. The Bcl-2 family protein Mcl-1 was expressed in normal cells, and it was discovered that miR-204 regulated gene expression in normal cells (Chen et al., 2013). miR-204 binds to the 3′ untranslated region (3′UTR) of the Mcl-1 encoding gene, hindering its expression. In TL-treated cells, Mcl-1 underwent a dose and time-dependent decrease, while miR-204 levels increased. By inhibiting the expression of Mcl-1, TL favors an increase in the mitochondrial membrane permeability.

One of the least understood mechanisms influenced by TL in PC cells is the inhibition of 5-lipoxygenase (5-LOX). 5-LOX is overexpressed in normal PC cells. One mechanism through which this occurs is the activation of Bcl-2 through leukotriene B4 (LTB4) signaling triggered by 5-LOX (Zhou et al., 2007). Bcl-2 works against Bcl-2 proteins, such as BAX and BAK, to keep the mitochondrial membrane less permeable. Bcl-2 has been shown to attenuate TL's pro-apoptotic effects when upregulated in PC cells (Wang et al., 2006). Upregulation of Bcl-2 can be caused by an increase in LTB4 or the upregulation of 5-LOX. Studies have suggested that TL acts to downregulate 5-LOX and LTB4 production in a dose- and time-dependent manner in PC cells (Zhou et al., 2007). The mechanism of action is not clearly understood because TL is given to cells in a dose- and time-dependent manner, thus, the amount of cellular Bcl-2 protein is not affected (Zhou et al., 2008), meaning that even though Bcl-2 can attenuate TL-induced apoptosis, and Bcl-2 can be upregulated by 5-LOX and LTB4, the downregulation of these pathway components has no effect on Bcl-2. This finding indicates Bcl-2 is being regulated by multiple pathways in PC cells and that some of these pathways are not affected by TL.

Another pro-apoptotic effect of TL through the downregulation of the nuclear factor kappa-light-chain-enhancer in activated B cells (NF-κB) and its effects on Bcl-2 family proteins. NF-κB has protumour and prometastasis effects related to cell survival, however, the most effect, in terms of an examination of TL, is its relation to Bx-cl and Bfl-1. Similar to Bcl-2 and Mcl-1, Bx-cl and Bcl-2 are pro-survival proteins that act by decreasing the permeability of the mitochondrial membrane. This act decreases the amount of cytochrome c released into the cytosol for use in apoptotic pathways (Wang et al., 2006). Totally, TL downregulates NF-κB and Bcl-2 proteins it transcribes through two different pathways.

One of the two mechanisms inhibited by TL that downregulate NF-κB is the alteration of the poly (ADP-ribose) polymerase-1(PARP-1) protein. PARP-1 is observed to be overexpressed in PC cells, increasing the resistance, proliferation and metastasis of PC. TL acts upon PARP-1 by cleaving it into an inactive form (Wang, 2012), which decreases the nuclear accumulation of NF-κB, downregulating Bx-cl and Bfl-1 (Wang et al., 2012).

The second mechanism that downregulates NF-κB is through the inhibition of specificity protein 1 (Sp1) by TL. Sp1 has significant effects beyond the transcription of NF-κB. Sp1 is a nuclear translocation of p50 and p65 of NF-κB. In PC, Sp1 is overexpressed, resulting in an upregulation of NF-κB. In TL treated PC cells, there was a marked decrease in Sp1 activity. However, the levels of Sp1 mRNA expression were not affected. This result points to either TL directly inhibiting Sp1 or an upstream event doing the same. The upstream action was found to be the biosynthesis pathway that regulates signaling and transcription factors. This pathway increases the activity of O-linked β-N-acetylglucosamine (O-GlcNAc) transferase, causing it to glycosylate targets at a faster rate. The Ser-484 of Sp1 is a target of O-GlcNAc transferase's glycosylation. The activates allowing Sp1 to bind to promoter sequences. Although the exact section of the hexosamine biosynthetic pathway (HBP) that is affected by TL in PC cells is unknown, the result is the inhibition of O-GlcNAc transferase activity. This inhibition leads to Sp1 not being glycosylated and not acting as a transcription factor of the p65 and p50 subunits of NF-κB (Banerjee et al., 2013).

The most complex mechanism through which TL increases mitochondrial membrane permeability is the inhibition of pathways surrounding heat shock protein 70 (HSP70). In PC TL treated cells, HSP70 was found to be downregulated, and protein and mRNA levels were decreased (Phillips et al., 2007). To determine whether this downregulation of HSP70 results in the induction of the UPR by TL, another investigation evaluated the effect on the PERK-eIF2α arm of the UPR (Mujumdar et al., 2014). And in PC cells, the inhibition of HSP70 only seems to affect apoptosis; autophagy is not affected by TL-induced downregulation of HSP70 (Wang et al., 2006). The mechanism through which HSP70 is activated in pancreatic adenocarcinoma cells shares similarities with the activation of NF-κB. HSP70 is partially transcribed by the Sp1 which is activated through the HBP. It increases O-GlcNAc glycosylation, leading to ser-484 in Sp1 to be glycosylated, thus activating it. This pathway is overexpressed leading to HSP70 also being overexpressed. Because of TL's effects on the HBP, Sp1 is inactivated thus HSP70 is downregulated. But TL works through HSP70 (Banerjee et al., 2013). Heat shock factor 1 (HSF1) is a transcriptional factor of HSP70 that binds to heat shock sequence elements (HSEs) throughout the genome. This process promotes the transcription of HSP. TL acts upon HSF1 by decreasing the mRNA and protein levels. This process also decreases the amount of HSF1/HSE binding throughout the genome, downregulating HSP70 (Wang et al., 2012). The inhibition of promoters is not the only manner in which HSP70 is downregulated. miRNA also plays a role in the inhibition of transcription. As in the case of the Bcl-2 family protein Mcl-1, miR-142-3, was found to decrease transcription of the DNA it bound to. miR-142-3 binds to 3′UTR of the HSP70 gene, regulating the levels of transcription. TL exposure induced miR-142-3 in pancreatic ductal adenocarcinomas, suggesting that TL has multiple effects on the miRNAomes of PC cells (Mackenzie et al., 2013). HSP70 has been shown to inhibit the activities of a pro-apoptotic protein and a pro-apoptotic ion, Ca^2+^ and cathepsin b, respectively. Because HSP70 is downregulated in TL-treated cells, there is less HSP70 available to attenuate Ca^2+^, which allows the levels of cytosolic Ca^2+^ to increase (Dudeja et al., 2009). HSP70 has been shown to decrease the permeability of lysosomes in a manner similar to the interaction between Bcl-2 and the mitochondrial membrane. By downregulating HSP70, TL increases the permeability of the lysosome, allowing cathepsin b to act upon the mitochondrial membrane (Dudeja et al., 2009). Mitochondrial membrane permeability is one of the most complex factors influenced by TL in PC cells. Through the up and down-regulation of Bcl-2 family proteins, transcription factors, proteases, ions and miRNAs, the pathway of apoptosis continues through the release of cytochrome c from within the mitochondria. Moreover, the combination of TL and artesunate (an anti-malarial natural product derivative for cancer therapy) could inhibit PC cell line growth and induce apoptosis, which is accompanied by the expression of HSP20 and HSP27, indicating important roles in the synergic effects in an *in vitro* study (Liu and Cui, 2013).

#### Autophagic pathways

Other mechanisms of cell death induced by TL have been explored. When PC cells underwent a knockdown of autophagy-specific genes atg5, cell viability was maintained after TL treatment, indicating that TL impacts the autophagic pathways of cell death (Borja-Cacho et al., 2010; Mackenzie et al., 2013). To find which pathways TL affects to decrease cell viability through autophagy, two pathways of nutrient starvation-induced autophagy were observed after TL treatment, include the Akt/mTOR/p70s6K and raf-1/Mek-1/ERK1/2 pathways. These pathways are common mechanisms through which autophagy is induced in cancer (Mujumdar et al., 2010). Treatment of autophagy-induction favoring S2-VP10 and S2-013 with TL showed downregulation of phosphorylated Akt and phosphorylated mTOR. This result shows that TL affects Akt either directly or through an upstream mechanism and that downregulation continues through the pathway, inhibiting a negative regulator of autophagy (Mujumdar et al., 2010).

The mechanisms through which TL promotes cell death have been reported to affect both autophagy and apoptosis. The Bcl-2 family protein Mcl-1 also acts to decrease autophagy-induced cell death in PC cells (Chen et al., 2013). The pro-apoptotic ion Ca^2+^ has also been related to increased activation of autophagy in PC cells (Mujumdar et al., 2010). It appears that TL activates autophagy by increasing mitochondrial membrane permeability, as well as affecting nutrition starvation-induced autophagic pathways.

#### Angiogenic pathways

TL has been shown to promote autophagy through starvation-induced autophagy. It has also been reported that TL plays a role in inducing nutrition starvation in PC cells through angiogenesis (Ma et al., 2013). In PANC-1, TL has been shown to inhibit angiogenesis through VEGF (Ding et al., 2015; Qiao et al., 2016). In TL-treated cells, the expression of VEGF and COX-2 was significantly decreased, as indicated by a decrease in the mRNA levels of these proteins. The sensitivity of this autophagic pathway is also increased by TL's effects on regulatory pathways, meaning that the autophagic signal would be much stronger (Ma et al., 2013).

## Discussion

Of the 116 articles initially examined, 17 were definitively included in the present study. Existing preclinical evidence suggests that TL has broad spectrum of anti-PC effects, including proliferation inhibition, apoptosis induction and metastasis inhibition. The pathways that TL alters to generate apoptotic, autophagic and angiogenic signals within cells are often the same pathways that provide PC cells with their extreme resistance to treatment. The overexpression of HSP70 and many other proteins has been connected in some manner to the attenuation of the pathways that occur in normal cells. As we know, many clinical medications, such as gemcitabine, have failed because of the extreme resistance exhibited by cells. Numerous preclinical studies remind us that if TL could be used in combination with clinical medication that hinders such resistance, then potent anticancer effects could occur (Wang, 2012; Qiao et al., 2016). One barrier to clinical trials is the reporting of standardized safety data from preclinical animal models.

Although TL has been reported to show anticancer effect for over a decade, its mechanisms of action have failed to attain one elusive goal. In the past 10 years, several groups have discovered that TL inhibited the activity of RNA polymerase. Due to the PC-based search strategy and inclusion criteria of this systematic review, we cannot include all breakthrough findings concerning the molecular target of TL. However, we cannot ignore these findings. One study has reported that TL inhibited the whole genome without affecting the binding of DNA and transcription factors. Investigators reported that TL inhibited the transcriptional activity of RNA polymerase II (RP II) and the degradation of Rpb1 and short-lived mRNA (Pan et al., 2010). Rbp1 binds with XPB to ensure transcription, confirming the mechanism through which TL inhibited gene transcription in cells (Titov et al., 2011; Wang et al., 2011). Rpb1 ubiquitination is regulated by a phosphorylation of the carboxyl-terminal-domain (CTD). In 2012, TL was confirmed to control RP II degradation through CDK7 (Manzo et al., 2012). Undoubtedly, TL can inhibit the transcriptional activity of RP II, inhibiting genome-wide transcription. However, this target requires further investigation in future research.

Although TL possesses a variety of bioactivities and pharmacological *in vivo* and *in vitro* effects, it has been restricted in clinical application due to its toxicity. Genome-wide microarray analyses suggest TL-induced toxicology shares a similar underlying mechanism with the pharmacological effects of TL (Li et al., 2014). The strong evidence of this potential connection is needed. To achieve lower toxicity with better efficacy, further research on the combinatorial effects with other drugs must be conducted, or bioinformational methods could be used.

**Figure 2:**
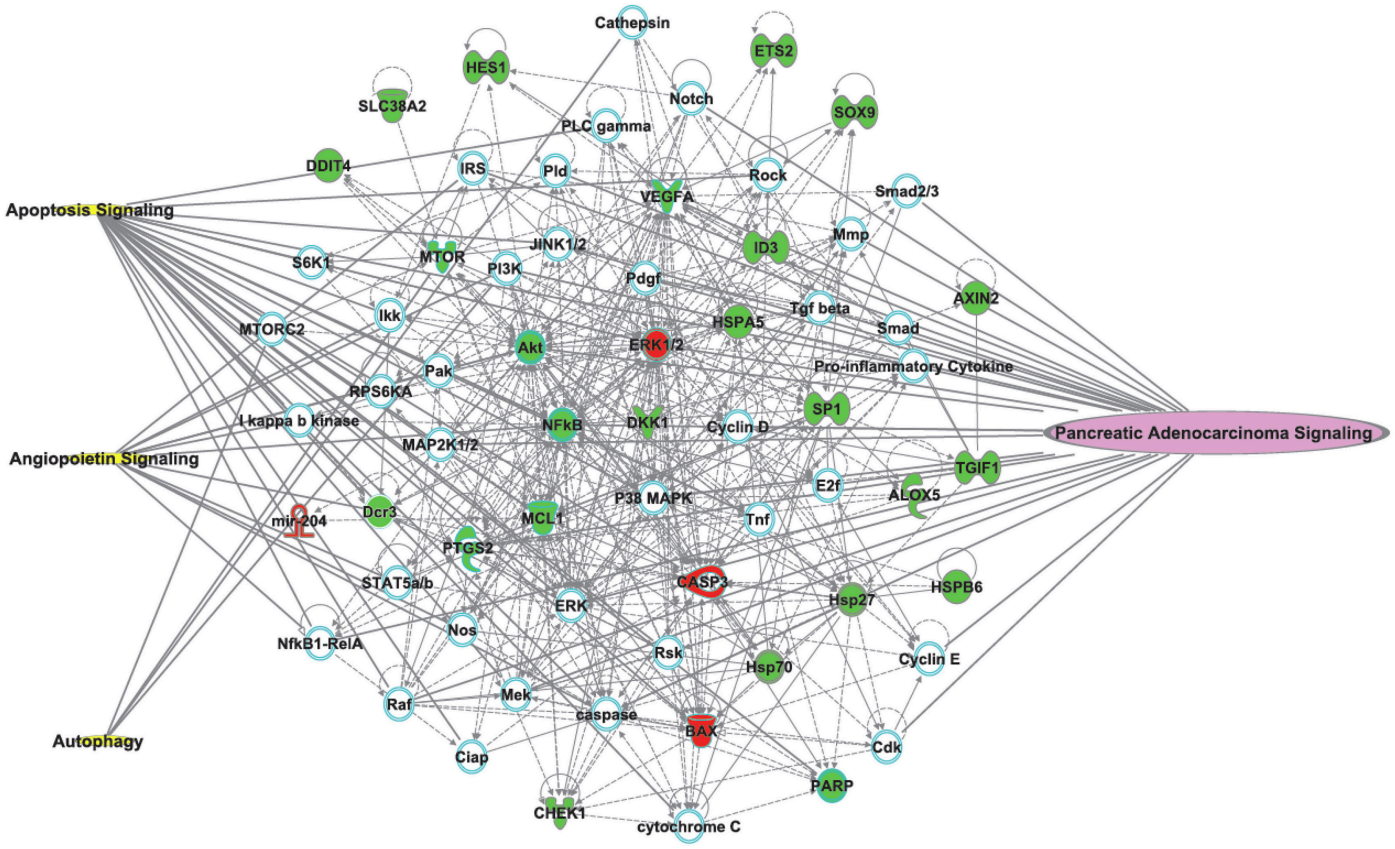
The most significantly relevant pathways and highly linked molecules (identified molecules from 17 preclinical studies which to explore the potential mechanisms of TLs for pancreatic cancer) relevant to the merged bionetwork. In the network, molecules are represented as nodes, and the biological relationship between two nodes is represented as a line. Red nodes and green nodes represent up- and down-regulated molecules, respectively. Yellow symbols represent three signaling pathways related to the merged bionetwork. One pathway on the right side of the bionetwork “Pancreatic Adenocarcinoma Signaling” indicates the highly linked molecules and pancreatic cancer signaling pathways. Solid lines between molecules show a direct physical relationship between molecules, whereas dash lines show indirect functional relationships.

To gain further insight into the molecular mechanism of TL for PC, we built a network using the Ingenuity® Pathways Knowledge Base (Ingenuity Systems Inc., Mountain View, CA, USA). The most significantly relevant pathways and highly linked molecules were built into a bionetwork (Figure 2). The identified molecules (red & green colored) are from 17 preclinical studies that explored the potential mechanisms of TL treatment for PC in our systematic review. They are the primary endpoints in these preclinical studies. The used pathway information exploits the signaling knowledge stored in public databases. Three signaling pathways are related to the bionetwork. Pancreatic adenocarcinoma signaling indicates the highly linked molecules and PC signaling pathways. Bioinformatics analysis was performed to identify the key molecules and signaling pathways relevant to TL and PC. Further study with bioinformatics will hopefully delineate the enriched pathways of TL for PC.

Note that the reported actions of TL were only observed in preclinical studies. TL has a major hurdle to overcome in its clinical use because it lacks water solubility. To rectify this weakness, a prodrug called Minnelide was developed (Chugh et al., 2012). Because of the available documentation (Banerjee et al., 2014, 2016; Nomura et al., 2015; Modi et al., 2016) discussing the biochemistry, effects and minimal toxicity of Minnelide, it is currently undergoing clinical trials. A phase II, open label trial of Minnelide™ is recruiting in patients with refractory PC (ClinicalTrials.gov Identifier: NCT03117920) now. The sample size is 35, and the estimated study completion date is February 2019.

Finally, as illustrated in Table 1, the results of negative preclinical studies are less likely to be published. These preclinical studies may have a bias toward an overestimation of favorable outcome. Randomization and blinding, the quality of preclinical studies, is typically reduced in comparison to clinical trials.

## Conclusion

In conclusion, based on preclinical evidence, TL's anti-pancreatic cancer profile has been shown in different experimental models and under different experimental conditions. The underlying mechanisms were summarized, with special emphasis on the apoptosis, autophagy, angiogenesis and related molecular pathways. In terms of treatment allocation methods reporting, randomization and blinding, improved study quality will accelerate needed progress toward more clinical investigations.

## Author contributions

Designers and writers: CZ and AL; Searchers and data extractors: CZ and XH; Studies appraisers; CZ, XH and all authors; Analyzers: CZ, XH, and LL.

### Conflict of interest statement

The authors declare that the research was conducted in the absence of any commercial or financial relationships that could be construed as a potential conflict of interest.

## Abbreviations

FasL: Fas Ligand
FasR: Fas Receptor
FADD: Fas-Associated protein with Death Domain
DISC: Death-inducing signaling complex
DcR3: Decoy Receptor 3
Bid: BH3 interacting-domain death agonist
tBid: truncated BH3 interacting-domain death agonist
MAC: Mitochondrial Apoptosis-Induced Channel
LTB4: Leukotriene B4
NF-κB: nuclear factor kappa-light-chain-enhancer of activated B cells
PARP-1: poly (ADP-ribose) polymerase-1
PC: pancreatic cancer
RA: rheumatoid arthritis
Sp1: specificity protein 1
SR: systematic review
TwHF: Tripterygium wilfordii Hook F.
